# Analysis of the Native Structure, Stability and Aggregation of Biotinylated Human Lysozyme

**DOI:** 10.1371/journal.pone.0050192

**Published:** 2012-11-16

**Authors:** Minkoo Ahn, Erwin De Genst, Gabriele S. Kaminski Schierle, Miklos Erdelyi, Clemens F. Kaminski, Christopher M. Dobson, Janet R. Kumita

**Affiliations:** 1 Department of Chemistry, University of Cambridge, Cambridge, United Kingdom; 2 Department of Chemical Engineering and Biotechnology, University of Cambridge, Cambridge, United Kingdom; 3 National Physics Laboratory, Teddington, United Kingdom; George Washington University, United States of America

## Abstract

Fibril formation by mutational variants of human lysozyme is associated with a fatal form of hereditary non-neuropathic systemic amyloidosis. Defining the mechanistic details of lysozyme aggregation is of crucial importance for understanding the origin and progression of this disease and related misfolding conditions. In this study, we show that a biotin moiety can be introduced site-specifically at Lys33 of human lysozyme. We demonstrate, using biophysical techniques, that the structure and stability of the native-state of the protein are not detectably altered by this modification, and that the ability to form amyloid fibrils is unchanged. By taking advantage of biotin-avidin interactions, we show that super-resolution fluorescence microscopy can generate detailed images of the mature fibrils. This methodology can readily enable the introduction of additional probes into the protein, thereby providing the means through which to understand, in detail, the nature of the aggregation process of lysozyme and its variants under a variety of conditions.

## Introduction

Since its discovery in the 1920's, lysozyme has increasingly emerged as an important system for studying protein structure and function [1], [2]. Members of the family of c-type lysozymes, which include the human form, are naturally occurring glycosidases involved in the degradation of bacterial cell walls. The hen protein was the first enzyme to have its structure defined at atomic resolution though X-ray crystallography and its study immediately shed important light on the mechanism of enzymatic catalysis [3]–[5]. The human form of lysozyme is a globular protein containing 130 amino acids and its native structure is typical of the c-type lysozymes [6]. It has four intramolecular disulphide bonds and two domains; the α-domain (residues 1–38, and 86–130), which consists of four α-helices (the A, B, C, and D-helix), and the β-domain (residues 39–85) containing a significant degree of β-sheet structure [6].

There have been many investigations of the folding mechanism of human lysozyme (see references within [7]). These studies became of particular interest following the discovery of two natural variants of lysozyme, I56T and D67H, which are linked to systemic amyloidosis. This fatal disorder is associated with the enhanced propensity of these naturally occurring mutational variants to self-assemble into amyloid fibrils [8]. This rare autosomal-dominant disease involves the accumulation of large amounts of fibrillar deposits in a wide range of tissues including the liver, spleen and kidneys [8]–[10], and is a member of a broader class of amyloid-related disorders, which include Parkinson's and Alzheimer's disease [11], [12]. Since the discovery of the I56T and D67H variants, four other naturally occurring disease-associated variants (F57I, F57I/T70N, W64R and T70N/W112R) have been identified, along with an additional variant (T70N) which is not disease-associated [13]–[16].

A range of detailed studies has been carried out to investigate the effects of these mutations on the *in vitro* folding and misfolding properties of human lysozyme. Relative to the wild-type (WT) lysozyme, the amyloidogenic variants, I56T and D67H, are characterised by a reduction in both the native state stability and the global co-operativity of the protein structure [9], [17]–[20]. These attributes result in the variants, but not the WT protein, being able to populate transient intermediate species under a variety of conditions, including those which are physiologically relevant, in which the β-domain and the C-helix are significantly unfolded, whilst the remaining parts of the protein maintain native-like structure [9], [18], [21]. The transient intermediate species can also be detected in the non-natural variant, I59T, under similar conditions and interestingly, they can be detected in both the T70N variant and WT protein under more highly destabilising conditions [22], [23]. The formation of a transient intermediate has been identified as a crucial step in lysozyme aggregation through studies which have made use of camelid antibody fragments to inhibit amyloid fibril formation [18], [24]. Fragments of two distinct camelid antibodies, cAbHuL-6 and cAbHuL-22, have been found to bind to the native state of WT lysozyme and the disease-associated variants with high affinity and to inhibit fibril formation by suppressing the formation of the transient intermediates [24], [25]. Along with the numerous *in vitro* studies, recent *in vivo* analysis shows that destabilising mutational variants of human lysozyme trigger the up-regulation of quality control mechanisms in *Pichia pastoris* and in *Drosophilia melanogaster* model systems [26], [27].

Given the wealth of data detailing the general mechanism of fibril formation by human lysozyme, it is of great interest to characterise this process at a molecular level, hence gaining structural details of the species present on this pathway. Recently, there have been notable advances in analysing and monitoring the early stages of amyloid formation using techniques such as single-molecule fluorescence measurements, fluorescence life-time imaging and multiparameter imaging microscopy [28]–[30]. The appearance, composition and structure of early oligomeric species of the fluorophore-labelled SH3 protein, the Aβ_1–42_ peptide and α-synuclein, have been the subject of detailed investigations performed *in vitro* [30]–[32]. Studies involving a fusion protein of α-synuclein and yellow fluorescent protein (YFP) have shown that α-synuclein-YFP maintains the properties of the unmodified protein to a remarkable degree. Indeed, using this system we have been able to define successfully key details of the kinetics of protein aggregation *in vivo* [31], [33].

To apply these methods to a protein system, it is essential to introduce a site-specific chemical probe into the protein. In some instances, fluorophores have been successfully incorporated via the introduction of single cysteine residues using site-directed mutagenesis, for example with the SH3 domain or α-synuclein [30], [34]. Lysozyme, however, contains eight cysteine residues that are involved in the formation of four disulphide bonds which help maintain and stabilise the native structure of the protein [35], [36]; the introduction of a chemical probe at a specific cysteine residue would, therefore, not be straight forward. Other attempts to modify lysozyme by introducing additional residues at both the C-terminus and N-terminus of the protein have been found to result in substantial perturbation to the native state stability of the protein [37], [38]. Recently, however, it has been reported that hen lysozyme could be labelled via the amine groups of lysine residues [39]–[41], however, the effects of the labels on the properties of the protein were not investigated in detail. Although multiple lysine residues are often present in proteins, it has been reported that the accessibility and mobility of individual lysine residues within proteins, coupled with strategies to control reaction conditions may result in the labelling of these residues in a selective manner [42], [43].

In this present paper, we describe the labelling of human WT lysozyme (WTHuL) with an amine reactive reagent, N-(+)-biotinyl-6-aminocaproic acid N-succinimidyl ester (BioNSE), as a strategy to incorporate site-specifically chemical reporters into the protein structure without significantly perturbing its native properties. Labelling with biotin derivatives has a number of important advantages; in particular, biotin has a strong specific affinity to avidin [44]. It can therefore be easily detected via streptavidin (SA) conjugates which are linked to a variety of reagents, for example, SA-alkaline phosphatase (AP) for colourimetric Western blotting or SA-fluorophores for fluorescence monitoring techniques [45], [46]. Using a number of biophysical techniques, we confirm in this work that the native structure and stability of the biotinylated human lysozyme (BioHuL) are comparable to WTHuL. In addition, we show that BioHuL can form fibrils *in vitro* and that we can monitor and visualise clearly, using super-resolution techniques, the fibrillar aggregates using streptavidin conjugated fluorophores and reagents.

## Materials and Methods

All chemicals were purchased from Sigma-Aldrich (Gillingham, UK) unless otherwise stated.

### Biotin-labelling of lysozyme by N-(+)-biotinyl-6-aminocaproic acid N-succinimidyl ester (BioNSE)

WT human lysozyme (WTHuL) (7 μM) was dissolved in MES buffer (100 mM, pH 5) with stirring in a glass vial (5 mL). BioNSE was freshly dissolved in DMSO (35 mM) immediately before use, and appropriate aliquots were added to protein solutions to give 1∶100 or 1∶500 lysozyme-to-BioNSE molar ratios. The samples were incubated with constant stirring for 20 h at room temperature and dialysed against deionised water (2×4 h). Mass spectrometry of the endpoint of the reactions indicated that the predominant species (∼80–90%) was singly-labelled WT lysozyme. Dialysed samples were flash frozen in liquid nitrogen and lyophilised. The samples were then dissolved in the respective buffers when required for further analysis.

### Purification of biotin-labelled lysozyme

Biotin-labelled lysozyme (BioHuL) samples were dissolved in Tris buffer (50 mM, pH 8) and purified using a CaptoS ion exchange column (GE Healthcare, Little Chalfont, UK) on an AKTA Prime Plus purification system (GE Healthcare). The protein was eluted using a sodium chloride gradient (0–1 M) and multiple fractions were collected. The fractions containing protein were dialysed against deionised water (2×4 h) and lyophilised. The protein integrity and purity were confirmed by mass spectrometry analysis and the final yield of purified protein, as determined by UV-vis spectroscopy was 55±5%. Samples containing the single biotin-labelled lysozyme were used in further experiments.

### Mass spectrometry

Both WTHuL and BioHuL samples were dissolved in deionised water and de-salted using ZipTips (Millipore, Watford, UK). Samples were analysed in triplicate on a 4700 Proteomics Analyzer (Applied Biosystems, Paisley, UK), using matrix-assisted laser desorption/ionization (MALDI) methodologies.

### Circular dichroism (CD) spectroscopy

CD spectroscopy experiments were performed using a Jasco J-810 spectropolarimeter (JASCO Ltd, Great Dunmow, UK) equipped with a Peltier temperature controller. WTHuL and BioHuL samples (20 μM) were dissolved in sodium citrate buffer (10 mM, pH 5) and analysed using a 0.1 cm or 1 cm path-length cuvette for far-UV or near-UV spectra, respectively. For secondary structure analysis, five spectra of each protein sample and of citrate buffer were recorded between 200 and 250 nm at 20°C (using a scan speed of 50 nm min^−1^ with a 1 nm band width and a 4 s response time). The spectra of the samples were averaged and corrected for the background buffer. Thermal denaturation was monitored at 222 nm or 270 nm as the temperature was increased monotonically from 5 to 95°C at a rate of 0.5°C min^−1^. Elipticity values were normalised to the fraction of unfolded protein (*F*_u_) using *F*_u_  =  (θ–θ_N_)/(θ_U_ – θ_N_), where θ is the observed ellipticity, and θ_N_ and θ_U_ are the ellipticities of the native and the unfolded states, respectively. θ_N_ and θ_U_ were extrapolated from pre- and post-transition baselines at the relevant temperature. Experimental data were fitted to a sigmoidal expression [47], using OriginPro 8.0 (OriginLab Corporation, Northhampton, MA, USA). Mid-point T_m_ values are defined as the temperatures where the *F*_u_ is 0.5 in each case.

### Nuclear magnetic resonance (NMR) spectroscopy

[^15^N]-labelled WTHuL was expressed in *Pichia pastoris* and purified as previously described [23]. Biotin-labelling of [^15^N]-labelled lysozyme was performed as described above for WTHuL. [^15^N]-labelled BioHuL or WTHuL (200 μM) was dissolved in sodium acetate buffer (20 mM, pH 5.0) containing a 90% H_2_O/10% ^2^H_2_O mixture. The solutions were filtered and sealed in a Shigemi tube, and data were collected at 37°C using a Bruker Avance 700 MHz NMR spectrometer equipped with a triple-resonance cryogenic probe. [^15^N]-[^1^H] heteronuclear single quantum coherence (HSQC) spectra were collected with 1024 and 128 complex points in t_1_ ([^1^H]) and t_2_ ([^15^N]), with sweep widths of 9470 and 2107 Hz in the [^1^H] and [^15^N] dimensions, respectively. All NMR spectra were processed with NMRpipe [48] and Sparky (http://www.cgl.ucsf.edu/home/sparky/).

### Proteolysis experiments

Proteolysis experiments involving the BioHuL and WTHuL samples were performed at the Cambridge Centre for Proteomics (Department of Biochemistry, University of Cambridge, Cambridge, UK). The samples were run on an SDS-PAGE gel and the bands were excised from the gel and transferred into 96-well PCR plates. The bands were cut into 1 mm^2^ pieces, destained, reduced with DTT and alkylated by treatment with iodoacetamide. The samples were subjected to enzymatic digestion with trypsin overnight at 37°C. After digestion, 10 μL aliquots of supernatant were put into sample vials and loaded onto an autosampler for automated LC-MS/MS analysis.

All LC-MS/MS experiments were performed using a nanoAcquity UPLC (Waters Corp., Milford, MA, USA) UPLC system and an LTQ Orbitrap Velos hybrid ion trap mass spectrometer (Thermo Scientific, Waltham, MA, USA). Separation of peptides was performed by reverse-phase chromatography at a flow rate of 300 nL/min, using a Waters column (BEH C18, 75 μm i.d. ×100 mm, 1.7 μm particle size).

The LC eluant was sprayed into the mass spectrometer by means of a New Objective nanospray source (New Objective, Inc., Woburn, MA, USA). All m/z values of eluting ions were measured in an Orbitrap Velos mass analyser, set at a resolution of 30,000. Data dependent scans (Top 20) were employed to isolate and generate fragment ions automatically by collision-induced dissociation in the linear ion trap, resulting in the generation of MS/MS spectra. Ions with charge states of +2 and above were selected for fragmentation and the data were processed using Protein Discoverer (version 1.2., Fisher Scientific UK, Loughborough, UK). All MS/MS data were submitted to the Mascot search algorithm (Matrix Science, London, UK) and searched against a custom human protein database, using a fixed modification of carbamidomethyl (cysteines) and variable modifications of oxidation (methionines) and biotin (lysines).

### Enzyme activity assay

50 μL aliquots of BioHuL or WTHuL (6.8 μM) were dissolved in potassium phosphate buffer (100 mM, pH 7) and placed in individual wells of a 96-well flat bottom microplate (Fisher Scientific UK). *Micrococcus lysodeikticus* cells (9 mg) were suspended in 30 mL of the potassium phosphate buffer shortly before the assay, and 200 μL of the cell suspension was added to each well [49]. Cell lysis was monitored at an absorbance of 595 nm after shaking and readings were recorded every minute over a 7 min interval on a FLUOstar Optima plate reader (BMG LABTECH, Aylesbury, UK). The results were averaged, plotted as a function of time, and then fitted linearly to calculate the slopes using OriginPro 8.0.

### Fibril formation of lysozyme samples monitored by light scattering or thioflavin-T binding

Aggregation studies were performed with BioHuL or WTHuL (6.8 μM, 3 M urea, 0.1 M sodium citrate buffer, pH 5.0, and 62.5 μM thioflavin-T (Thio-T)) incubated with stirring at 60°C in a Cary Eclipse spectrofluorimeter (Agilent Ltd., Oxford, UK). Thio-T fluorescence was measured with an excitation wavelength of 450 nm (slit width 5 nm) and monitored at an emission wavelength of 480 nm (slit width 5 nm). Fibrils for further experiments were prepared under similar conditions but in the absence of Thio-T; aggregation was monitored by light scattering at 500 nm with slit widths of 5 nm. All experiments were performed in triplicate.

### Seeded aggregation reactions

BioHuL or WTHuL fibrils were prepared as described above in the absence of Thio-T. The endpoint fibrils were sonicated using a Bandelin Sonoplus probe sonicator (Bandelin, Berlin, Germany), which was set at the minimum power level, pulsing 10% of the time over a five minute period. Aliquots of the sonicated fibrils (10% v/v) were added at the beginning of an aggregation time course reaction (6.8 μM, 3 M urea, 0.1 M sodium citrate buffer, pH 5.0, and 62.5 μM Thio-T) and the effects were monitored by changes in Thio-T fluorescence. Seeding experiments were monitored for WTHuL with the addition of BioHuL fibril seeds or WT fibril seeds and for BioHuL in the presence of WTHuL fibril seeds or BioHuL fibril seeds.

### Transmission electron microscopy (TEM)

Fibril samples (5 μL) collected at the endpoint of the aggregation reactions were applied to Formvar-coated nickel grids. The samples were stained with 2% (w/v) uranyl acetate, and imaged using a FEI Tecnai G_2_ transmission electron microscope (Multi-Imaging Unit in the Department of Physiology, Development and Neuroscience, University of Cambridge, UK) Images were analysed using the SIS Megaview II Image Capture system (Olympus).

### Dot blot assays

Samples taken from the endpoint of the aggregation reactions were centrifuged (16,000 x *g*, 10 min) and the supernatants removed. The pellets were washed three times with dH_2_O followed by centrifugation. The rinsed fibrils were resuspended in dH_2_O, (20 μL) and immobilised onto a nitrocellulose membrane (Whatman International Ltd., Maidstone, UK). The membrane was blocked with BSA (1% in phosphate buffered saline (PBS), 2 h), followed by incubation with streptavidin conjugated to alkaline phosphatase (AP) (Life Technologies, Paisley, UK) for 1 h. The membrane was washed three times with dH_2_O and developed using a 5-bromo-4-chloro-3-indolyl phosphate/nitroblue tetrazolium (BCIP/NBT) solution.

### Measurement of fibril stabilities

To measure the stability of fibrillar BioHuL and WTHuL, aliquots of fibrils were diluted into 0.1 M citrate buffer (pH 5.0) containing increasing concentrations of guanidinium hydrochloride (GdnHCl). After 48 hrs at 25°C, the samples were centrifuged (16,000× *g*, 15 min) and the concentration of lysozyme in the supernatant was measured by recording the absorbance at 280 nm. The depolymerisation curves were obtained by plotting the fraction of lysozyme released from the fibrils at different concentrations of GdnHCl. The final concentration of GdnHCl was determined by refractometry using [GdnHCl]  = 57.147(Δ*n*)+38.68(Δ*n*)^2^−91.60(Δ*n*)^3^, where Δ*n* is the difference between the refractive indices of GdnHCl solution and the 0.1 M citrate buffer [50].

### *d*STORM imaging experiments

Samples of BioHuL fibrils (10 μL) were placed on cover glass slips (18 mm ×18 mm, 170±5 μm thickness; Carl Zeiss Ltd., Welwyn Garden City, UK) and incubated with streptavidin-Alexa 647 (0.2 μg/mL) (Life Technologies Ltd.) for 1 h, followed by washing three times with PBS buffer. The switching buffer, composed of PBS (pH 7.4) containing an oxygen scavenger (0.5 mg mL^−1^ glucose oxidase, 40 mg mL^−1^ catalase (Roche Applied Science, Welwyn Garden City, UK), 10% w/v glucose) and 50 mM β-mercaptoethylamine (MEA), was prepared as previously described and loaded into a single cavity (15 mm diameter) glass microscope slide (Fisher Scientific UK) [51]. The cover glass with the fibrils was gently placed on the glass slide with the fibrils in contact with the switching buffer; bubbles were removed and the sample sealed with nitrocellulose solution to prevent evaporation. Super-resolution images were captured using a modified Nikon TE200 inverted total internal reflection (TIRF) microscope (Nikon Ltd. UK, Kingston upon Thames, UK). Fibre coupled diode lasers operating at 642 nm (150 mW) (Toptica Photonic AG, Graefelfing, Germany) and 405 nm (120 mW) (Mitsubishi Electronics Corp., Tokyo, Japan), were used as excitation and reactivation lasers, respectively. The laser beams were expanded and focused into the back focal plane of a 100X oil-immersion objective (Nikon UK Ltd.) BioHuL fibrils were imaged in TIRF mode for best contrast. A filter set containing a quad-edge laser flat dichroic beamsplitter (Semrock, Rochester NY, USA) and two emission filters (Semrock) were applied to eliminate straight fluorescent and excitation light. Typically, 10,000 image frames with an exposure time of 50 ms and field of view of 64×64 pixels were captured using a low-noise, highly sensitive electron-multiplying CCD camera (Andor iXon 897, Belfast, UK). The exposure time was matched with the ON state time of the fluorescent Alexa Fluor© 647 dye [52]. During data acquisition the intensity of the excitation laser on the sample was 1.5 kW/cm^2^ and the reactivation laser was only turned on when the number of active fluorophores in the field of view dropped, and no spatial drift of the sample was observed during the measurements. From the captured and stored image stack, a reconstructed *d*STORM image was generated in each case using in-house developed software based on Matlab (The MatWork Inc., Natick, USA). The software sequentially segments and localises isolated fluorescent molecules on each image frame and reconstructs the final super-resolved image. The localisation precision was determined on the basis of the number of photons captured per molecule [53].

### *d*STORM imaging in SH-SY5Y cells

Human neuroblastoma cells (SH-SY5Y) were obtained from the European Collection of Cell Cultures (Sigma-Aldrich, Dorset, UK) and grown in 1∶1 minimal essential medium (MEM) and nutrient mixture F-12 Ham (Sigma) with sodium bicarbonate, including 15% heat inactivated foetal bovine serum, 1% MEM non-essential amino acids, 2 mM N-glutamine, 1% penicillin-streptomycin (10,000 U ml K1) and 0.1% fungizone (amphotericin B, 250 mg mL^−1^ K1) (Life Technologies Ltd.).

Cells were harvested using trypsin (0.5 g L^−1^ of trypsin) with EDTA (0.2 g L^−1^ of EDTA 4Na) in Hanks′ Balanced Salt Solution (Life Technologies Ltd.), rinsed with growth medium and divided into 1×10^6^ cells in each 1.7 cm^2^ polystyrene chamber slide well (Lab-Tek^TM^ II, Fisher Scientific UK, Ltd.). Cells were subsequently transfected using an Amaxa nucleofector (Lonza, Cologne, Germany) with 35 ng of BioHuL fibrils in nucleofection buffer (Lonza).

Twenty four hours after electroporation the cells were washed three times with PBS, fixed for 10 min with formaldehyde (4% in PBS) before washing the cells three times with PBS. Tween (0.5% in PBS) was applied to the cells for 10 min for permeabilisation, followed by washing three times with PBS. After permeabilisation, the cells were incubated with Streptavidin-Alexa 647 (2 ng mL^−1^, Life Technologies Ltd.) for 30 min and washed three times with PBS. Switching buffer, as detailed above for *in vitro* super-resolution imaging, was added prior to imaging the cells. The samples were imaged by differential interference contrast (DIC) microscopy followed by single-molecule super-resolution imaging as detailed above.

## Results

### Site-specific biotin labelling of WT human lysozyme

**Figure 1:**
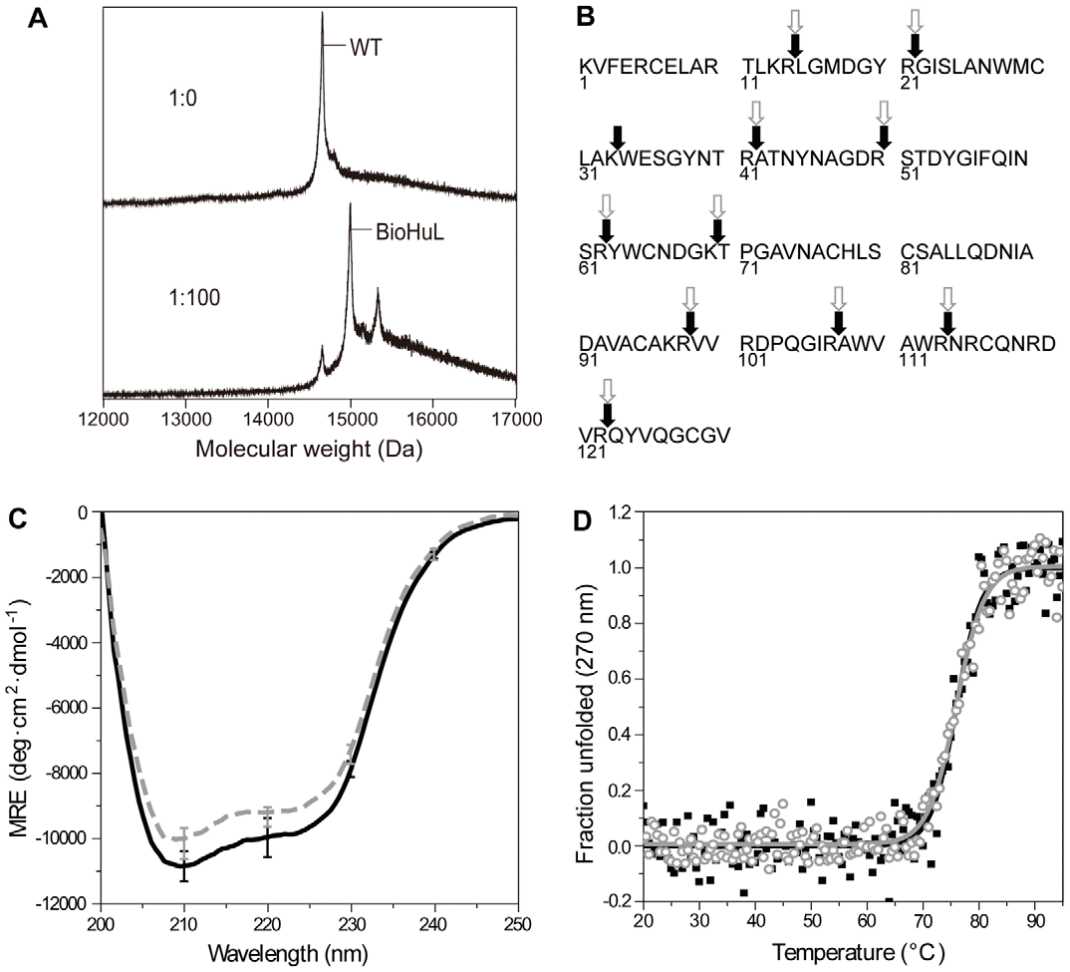
Effects of biotinylation on lysozyme. (A) MALDI mass spectrometry of lysozyme incubated in the absence (upper) and the presence (lower) of 100-fold molar concentration of BioNSE. (B) Primary sequence of human lysozyme showing the sites where trypsin digestion has occurred in BioHuL (open grey arrows) and WTHuL (filled black arrows). (C) Secondary structure of BioHuL (dashed grey line) and WTHuL (solid black line) by far-UV CD. (D) Thermal denaturation curves of BioHuL (open grey circles) and WTHuL (filled black squares) monitored by near-UV CD. Mid-point T_m_ values are defined as the temperatures at which 50% of the population of protein molecules is unfolded.

WT human lysozyme was incubated with different molar ratios of BioNSE and the reactions were analysed using MALDI mass spectrometry (Figure 1A). We observed that by incubating WT lysozyme overnight at room temperature with a 100-fold molar excess of BioNSE, the predominant resulting species is WT lysozyme modified with a single biotin moiety (BioHuL), displaying a peak at 15,033±1.5 Da. When the reaction was performed with a 500-fold excess of BioNSE, a less intense second peak at 15,372±1.5 Da becomes evident, corresponding to the incorporation of two biotin moieties within WT lysozyme. Using ion exchange chromatography, the singly-labelled BioHuL protein, formed following incubation under these conditions, was purified from both the unmodified protein and the doubly-labelled lysozyme.

To identify the site of the modification, the singly-labelled BioHuL sample was subjected to trypsin proteolysis and analysed by mass spectrometry. The analysis reveals that the singly-labelled BioHuL displays a substantial change in the tryptic digestion pattern, and that modification of Lys33 results in the loss of a trypsin cleavage site. This results in the appearance of a longer peptide fragment (residues 22–41) in BioHuL than the fragment observed in WTHuL (residues 22–32) (Figure 1B) [54]–[56]. Despite WT human lysozyme having five lysine residues (Lys1, Lys13, Lys33, Lys69 and Lys97) which, according to the X-ray structure, all have side-chain amine groups that are highly solvent accessible and thus, in principle, able to react with an N-hydroxy-succinimide (NHS) ester group to which a chemical probe is attached, we have demonstrated that site-specific labelling of just one of these residues, Lys33, can be achieved. Previous reports of labelling lysine residues by a variety of techniques in hen lysozyme reveal that Lys33 again appears to be the most reactive lysine residue [41], [57]–[59].

### Effect of biotinylation on protein structure and native-state stability

Although it is possible to label WT human lysozyme site-specifically, there are no data available as to whether this modification affects the structural properties of the protein. We therefore used a number of biophysical techniques to compare the BioHuL protein, generated as described above, with that of the unlabelled WTHuL protein. Far-UV CD spectroscopy, which monitors the secondary structure change within the protein, shows that the secondary structure content of BioHuL is closely similar to that of the WTHuL (Figure 1C). Near-UV CD, which reports on tertiary structural changes within the protein, reveals that BioHuL and WTHuL have essentially identical thermal stability with T_m_ values of 76.0±0.5°C and 75.9±0.5°C, respectively (Figure 1D). T_m_ values of BioHuL and WTHuL obtained by far-UV CD also show comparable thermal stability data (data not shown). Given that BioHuL is structurally similar to WTHuL, we compared the enzyme activity of the two proteins by measuring their ability to lyse *Micrococcus lysodeikitcus* cells [49]. The BioHuL protein retains about 60% enzymatic activity as compared to WTHuL.

**Figure 2:**
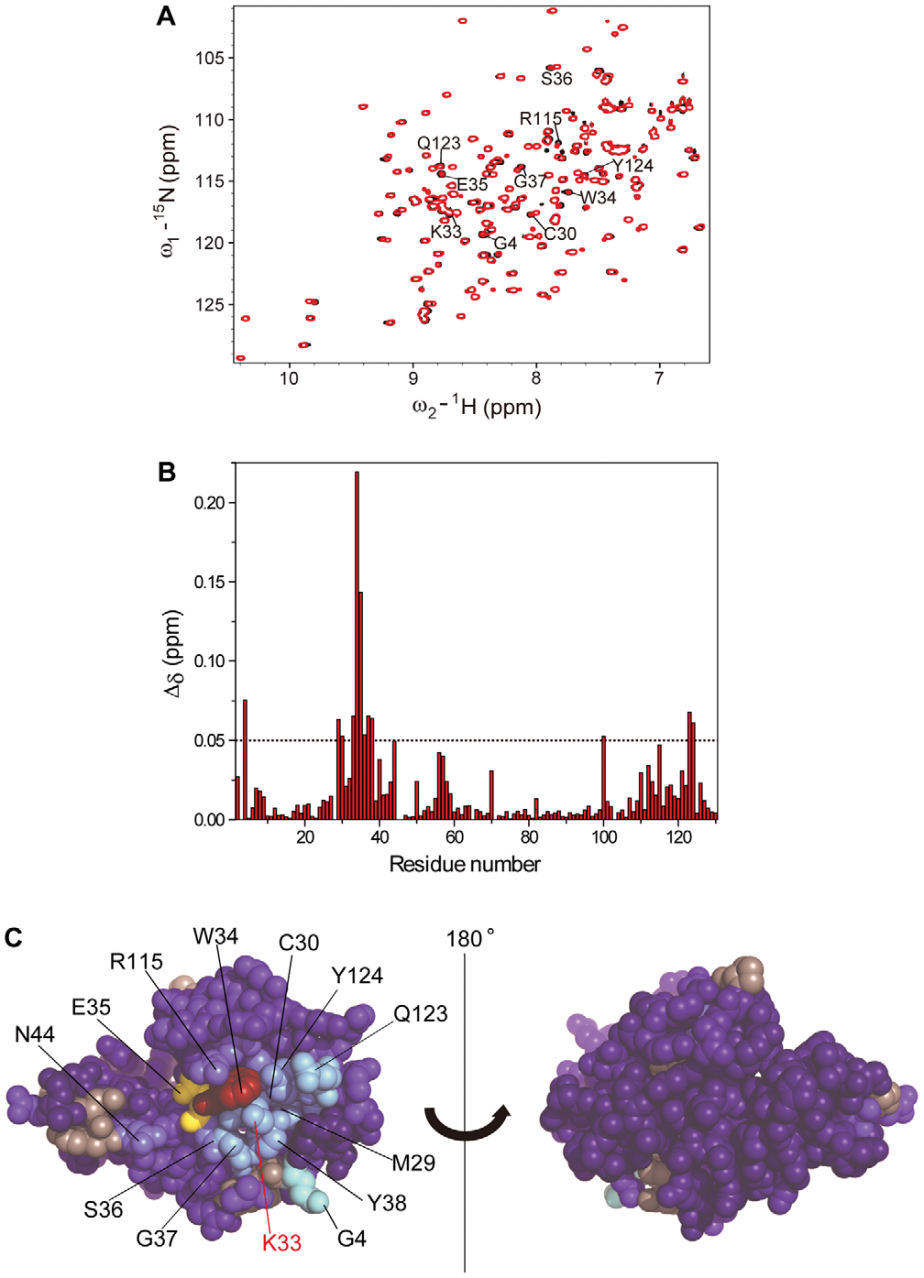
Mapping the location of modification by NMR spectroscopy. (A) Overlaid HSQC NMR spectra at 700 MHz of BioHuL (red) and WTHuL (blue); the spectra were collected at pH 5.0 and 37°C (B) Chemical shift changes defined as [0.04(δ^15^N_WT_−δ^15^N_Biotin_)^2^+(δ^1^H_WT_−δ^1^H_Biotin_)^2^]^1/2^ in BioHuL with respect to WTHuL [68]. (C) Structural identification of the BioHuL protein colour-coded by Δδ value of each residue observed in (B) with the modified Lys33 labelled in red. Dark blue represents the lowest Δδ value, whilst red represents the highest. Residues whose chemical shifts are most perturbed by the modification are identified. The black line in the centre of the two images represents the axis of rotation by 180°.

In order to understand the effects of the biotin-labelling on the protein at the level of individual residues, we compared the ^15^N-^1^H heteronuclear single quantum coherence (^15^N-^1^H HSQC) NMR spectra of BioHuL and WTHuL (Figure 2). In general, the parameters defining the backbone amide resonances of BioHuL are closely similar to those of WTHuL (Figure 2A). The chemical shifts (Δδ values) in particular, were compared closely (Figure 2B), and are shown mapped onto the structure of human lysozyme (Figure 2C). This comparison shows that the residues most affected by the modification are Trp34 (W34) and Glu35 (E35); the latter residue acts as an essential catalytic acid/base in lysozyme enzyme activity [5]. A combination of steric hindrance due to the biotin moiety preventing substrate binding, along with the perturbation to Glu35 due to the modification at residue 33, may provide some explanation for the slight decrease in overall enzymatic activity of BioHuL as compared to WTHuL. Although some minor perturbations were observed in residues that are spatially close to Lys33 (K33) (Figure 2C), the majority of Δδ values remained below 0.05 ppm indicating that there are no detectable perturbations of the signals of residues adjacent to other lysine residues. The chemical shift data therefore further supports the conclusion that the site of modification is indeed Lys33.

### Biotinylated lysozyme forms fibrils similar to WT lysozyme

**Figure 3:**
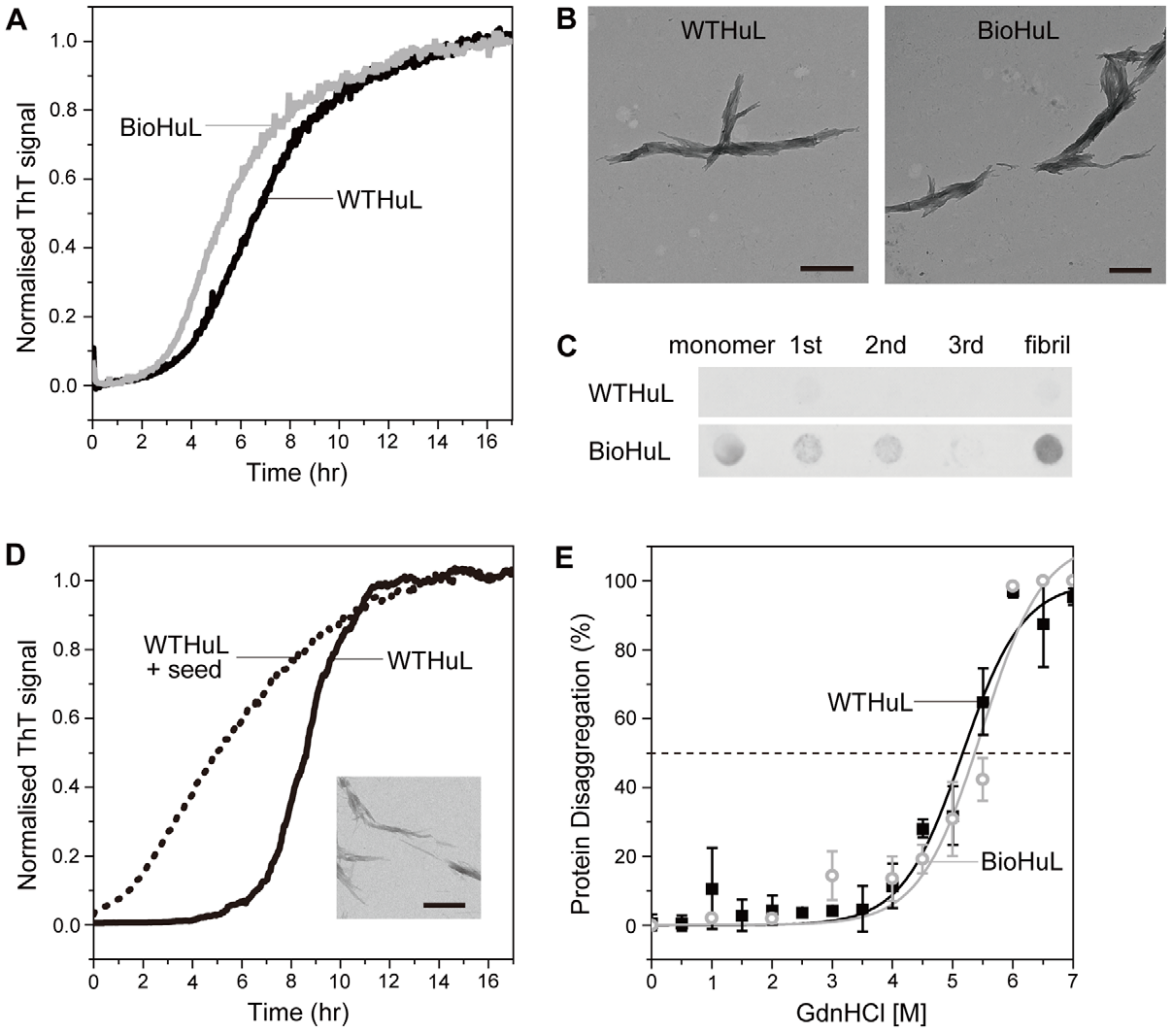
Fibril formation of BioHuL. (A) *In situ* Thio-T binding fluorescence kinetics of BioHuL (grey) and WTHuL (black) incubated in the presence of 3 M urea in 0.1 M citrate buffer (pH 5.0) with constant stirring at 60°C. (B) TEM images of the endpoint samples of the aggregation reactions performed in the absence Thio-T. Images on the left and right show the fibrils formed by WTHuL and BioHuL, respectively; the scale bars represent 500 nm. (C) Dot blot assay of fibrils formed by WTHuL (upper row) and BioHuL (bottom row). Samples include the monomeric protein, the supernatant solutions after washing the fibril pellets (1^st^, 2^nd^, and 3^rd^) and the final washed fibrils. The appearance of colour indicates a positive interaction between biotin and streptavidin-AP. (D) *In situ* Thio-T binding fluorescence kinetics of WTHuL in the absence (solid line) and presence of 10% BioHuL fibril seeds (dashed line) incubated under similar conditions as (A). (Inset) TEM images of the WTHuL fibrils formed in the presence of BioHuL fibril seeds; the scale bar represents 500 nm. (E) Conformational stability of BioHuL (grey open circles) and WTHuL (black squares) fibrils. The stability of the fibrils was measured by depolymerisation experiments performed using GdnHCl at pH 5.0. Continuous lines represent the best fits to sigmoidal functions.

Our analysis has revealed that BioHuL possesses similar native-state properties to that of WTHuL. We next investigated the misfolding and formation of fibrillar species by BioHuL. Amyloid fibrils have been shown to be formed *in vitro* from WT lysozyme under a number of different solution conditions that involve destabilisation of the native-state, notably by the use of elevated temperatures at low pH or in the presence of chemical denaturants [24], [60]–[62]. Here, we incubated both BioHuL and WTHuL proteins at 60°C in 0.1 M citrate buffer (pH 5.0) in the presence of 3 M urea and monitored, in each case, enhancement of thioflavin-T (Thio-T) fluorescence to follow the formation of ordered amyloid aggregates. Both proteins were observed to form fibrils after a lag phase of approximately 4 hours and displayed typical sigmoidal curves (Figure 3A). The endpoint samples were analysed by transmission electron microscopy (TEM), and the resulting images confirmed that the size and morphology of the fibrils formed by BioHuL are effectively identical to those formed by WTHuL. In addition, these fibrils clearly resemble those formed in previous studies where WTHuL fibrils were generated at pH of 7.5 (60°C) [62]; in each case, the fibrils appear bundled and intertwined together with lengths around 1-2 μm and about 100 nm in thickness (Figure 3B).

Although the endpoint fibrils appear very similar in the TEM images, we also investigated whether the fibrillar seeds formed from BioHuL and WTHuL could cross-seed the aggregation reactions of WTHuL and BioHuL respectively. Indeed, the addition of 10% (v/v) preformed fibril seeds is highly efficient at promoting aggregation in the cross-seeding reactions which suggests that the fibrils formed in both instances have similar morphologies (Figure 3D). In order to assess the conformational stability of the BioHuL and WTHuL fibrils, we measured the resistance of the fibrils to depolymerisation by incubating fibril aliquots in solutions containing guanidinium hydrochloride (GdnHCl) and measuring the concentration of soluble monomer released into the supernatant. The plots of the fraction of soluble protein present at increasing GdnHCl concentrations are shown in Figure 3E. Both the BioHuL and WTHuL fibrils have similar midpoints of depolymerisation, 5.5±0.5 M and 5.2±0.3 M respectively, therefore demonstrating similar conformational stability.

We next set out to determine if the biotin moiety remains solvent accessible in the BioHuL fibrils formed in 3 M urea at pH 5.0, by using a dot blot assay; by taking advantage of the very high affinity of biotin for the protein, streptavidin [44], we used streptavidin conjugates to probe for the presence of biotin in the BioHuL fibrils. In this assay, the fibrils were washed and immobilised onto nitrocellulose and the membrane was then probed with a streptavidin-alkaline phosphatase conjugate; alkaline phosphatase activity was then measured, resulting in a colourimetric reaction. To ensure that the centrifugation and washing of the fibrils did not alter the morphology, the structures of the samples were confirmed by TEM imaging and no changes were observed. Both the monomer and fibrils of BioHuL give a positive signal for the presence of biotin (Figure 3C), showing that the biotin-moiety is solvent exposed in the fibrils and therefore can, in principle, act as a useful tag for characterising the species formed during aggregation.

**Figure 4:**
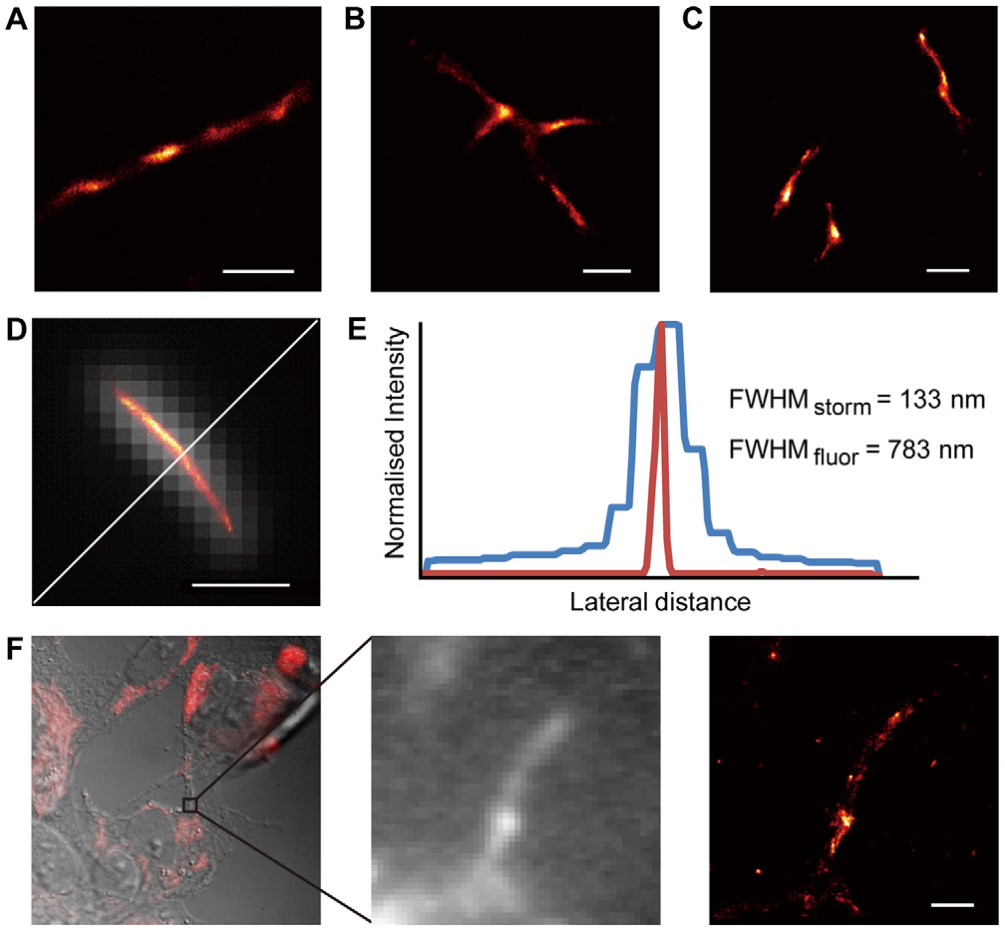
*d*STORM images of BioHuL fibrils. (A)–(C) Super-resolution *d*STORM images of different BioHuL fibrils (formed *in vitro*). (D) An overlay of a straight BioHuL fibril with its fluorescence sum image. (E) The cross-sections of the individual fluorescence sum and the super-resolved *d*STORM image of the BioHuL fibril displayed in panel (D). The full-width half-maximum (FWHM) of the fluorescence intensity distribution of the unresolved sum image depicts a fibril diameter of 783 nm whereas the super-resolved image depicts a fibril diameter of 133±20 nm; the latter showing 6 times better resolution. (F) (left panel) DIC image of BioHuL present within SH-SY5Y mammalian cells after probing with streptavidin-Alexa647. (middle panel) Fluorescence sum image of a region within the SH-SY5Y cells (right panels) Super-resolution *d*STORM images of BioHuL fibrils in the same region of the SH-SY5Y mammalian cells as the fluorescence sum image. All scale bars all represent 1 μm.

Finally, we labelled the BioHuL fibril samples with streptavidin-Alexa647 and imaged them directly using direct stochastic optical reconstruction microscopy (*d*STORM); this super-resolution imaging technique can provide information on spatial scales as low as 20 nm, much smaller than the wavelength of the probing light [52]. In a related study we have shown that *d*STORM can be used to identify structural differences between aggregated states of fluorescently-labelled Aβ_1–42_ peptide formed both *in vitro* and in cells [63]. The *d*STORM analysis of the BioHuL aggregates clearly reveals fluorescent fibrils with greatly improved resolution compared to that achievable with conventional fluorescence techniques (Figure 4). Figure 4D shows a fibril bundle with a diameter of 133±20 nm, which is comparable to the diameter observed in the TEM images of similar bundles (approximately 100–200 nm) (Figure 3B).

*d*STORM not only offers high resolution structures of single fluorophore labelled samples, but it offers the compelling advantage of using multiple fluorophores which allows the imaging of various proteins simultaneously and selectively [64]. Another great advantage provided by *d*STORM is that it is possible to apply this technique *in situ* thereby allowing direct imaging of species within the cellular environment. To demonstrate this capacity, we introduced BioHuL fibrils, prepared by the *in vitro* methods, into SH-SY5Y mammalian cells using an electroporation technique previously reported [33]. After washing the cells and permeabilising them with 0.5% v/v Triton-X 100, the streptavidin-Alexa647 conjugate was added. Differential interference contrast (DIC) microscopy imaging confirms that the BioHuL fibrils are present inside the SH-SY5Y cells (Figure 4F, left panel) and a traditional fluorescence image is shown (Figure 4F, middle panel) along with a subsequent *d*STORM image of the BioHuL fibrils inside the SH-SY5Y cells (Figure 4F, right panel). As amyloidogenic lysozyme fibrils are found in patients as extracellular deposits [8], it is interesting that the fibrils introduced to the intracellular environment appear somewhat the same in structure to those observed in the *in vitro* images in Figure 4A–C. Although much further work is needed to understand the significant of these preliminary data, the results demonstrate that super-resolution imaging of biotin-labelled lysozyme has real potential for characterising the process of fibril formation, and therefore for investigating the effect of potential modulators of fibril formation both *in vitro* and in a cellular environment.

## Discussion

In this study, we have successfully introduced a site-specific biotin label into WT human lysozyme and have shown that this modification has no significant effects on native-state structure and stability or on the process of *in vitro* fibril formation by the biotinylated lysozyme. It has been demonstrated in previous studies that the regions of human lysozyme containing the β-domain and the C-helix in the native state are unfolded in the transiently partially unfolded intermediate which is crucial for fibril formation [24], [65]. The location of Lys33 is within the B-helix, on a solvent exposed face of the α-domain in the native state of human lysozyme, and this region is not directly involved in the formation of the transient intermediate; therefore, it is consistent that modification to this residue does not result in significant changes to the process of *in vitro* fibril formation. The position of the biotin modification has the great advantage that we are able to probe the BioHuL fibrils formed at pH 5.0 via a streptavidin-AP conjugate; our results show that the biotin moiety resides on an exposed surface, and thus in an accessible region of the fibrils. We exploit this property to obtain fluorescence microscopy images of the mature fibrils, both in solution and following introduction into SH-SY5Y mammalian cells, at very high resolution using the *d*STORM super-resolution imaging technique.

The availability of the biotin moiety located on Lys33 for detection with streptavidin conjugates provides strong evidence that the size of the fibrillar core of these fibrils more closely resembles that of WTHuL fibrils formed at pH 7.5, 60°C than to those formed at pH 1.5, 45°C [60], [62]. For the latter, on the basis of limited proteolysis experiments, the fibrillar core has been suggested to consist of residues 32–108 [60] and FTIR analysis confirms that 75% of the sequence exists as β-sheet structure. In contrast, fibrils formed at the higher pH were found to have a different morphology, and although the core region has not been defined in detail by limited proteolysis experiments, it has been found to consist of a distinctly smaller β-core region, with 45% of the sequence in β-sheet structure [62]. The location of a chemical probe at Lys33 should be very useful for monitoring conformational changes throughout the process of fibril formation at physiologically relevant pH values. In addition, ready availability of a variety of succinimide ester-conjugated probes will enable us to use the site-specific modification technique described in this study to introduce a wide range of chemical species into human lysozyme, opening up the prospects for a wide range of studies involving, for example, spin-labelling NMR and single-molecule fluorescence experiments [32], [52], [66], [67]. These studies should allow us to gain insight into the molecular details of species present along the fibril forming pathway of human lysozyme, and hence, to describe in molecular detail, the mechanism of this extremely important disease-associated process.
